# Supplementary material for: A compact co-aperture dual-sense circularly polarized antenna for simultaneous transmit and receive systems
Source: PLoS One. 2024 Jul 5;19(7):e0304414. doi: 10.1371/journal.pone.0304414 (PMC11226108; doi:10.1371/journal.pone.0304414)
Supplement: Attachment — Submitted filename: Comments to the Author.docx [file pone.0304414.s001.docx]

**Title:** A compact co-aperture dual-sense circularly polarized antenna for simultaneous

transmit and receive systems.

**Manuscript Number:** PONE-D-24-05575

**Authors:** Duc-Nguyen Tran Viet, Dat Nguyen Tien, Dinh Nguyen Quoc, Tung The-Lam Nguyen, Hung Tran-Huy, Ph.D.

Comments to the Author

This paper proposes a compact design of dual-sense circularly polarized for simultaneous transmit (Tx) and receive (Rx) communication systems. The high Tx-Rx isolation can be achieved by tuning the feeding positions. There are some major issues that raise my concerns.

1. Undoubtedly, the isolation of the transmitting and receiving ports is a critical parameter for a STAR antenna. Although the article explains how to achieve high Tx-Rx isolation by merely adjusting the feed position, its decoupling mechanism is not explicitly described. To enhance the reader's comprehension, kindly provide a comprehensive description of this. Also, could you please state what challenges there are for the decoupling problem of dual circularly polarized antennas with the same aperture?

2. In contrast to the co-aperture dual-sense CP antennas that are comparable in size, the antenna proposed in the article possesses the most limited bandwidth. Could you please provide an explanation as to why?

3. More comprehensive results need to be provided so that readers can clearly understand the effectiveness of the proposed antenna, such as antenna efficiency, envelope correlation coefficient, and radiation pattern of both ports.

4. In Figure 12, it can be clearly observed that the tested S21 is better than the simulated one. Is this phenomenon coincidental? If not, then elucidate this.

5. There are many grammar mistakes in this paper. For example, The last paragraph of the introduction. "In this paper, the dual CP antenna with size miniaturization is concentrated. The proposed **STR** antenna employs 2 ×2 unit-cell MS as the co-aperture to achieve compact size. ". Page 4, third line of the first paragraph, "Fig. 7shows the geometry of the proposed dual-sense CP antenna." Please add a space after 7
